# Retrospective Attention Gates Discrete Conscious Access to Past Sensory Stimuli

**DOI:** 10.1371/journal.pone.0148504

**Published:** 2016-02-10

**Authors:** Louis Thibault, Ronald van den Berg, Patrick Cavanagh, Claire Sergent

**Affiliations:** 1 Laboratoire Psychologie de la Perception (UMR 8242), Université Paris Descartes, Centre National de la Recherche Scientifique, 75006 Paris, France; 2 Judgment and Decision Making Group, Department of Psychology, University of Uppsala, 751 05 Uppsala, Sweden; Radboud University Nijmegen, NETHERLANDS

## Abstract

Cueing attention after the disappearance of visual stimuli biases which items will be remembered best. This observation has historically been attributed to the influence of attention on memory as opposed to subjective visual experience. We recently challenged this view by showing that cueing attention after the stimulus can improve the perception of a single Gabor patch at threshold levels of contrast. Here, we test whether this retro-perception actually increases the frequency of consciously perceiving the stimulus, or simply allows for a more precise recall of its features. We used retro-cues in an orientation-matching task and performed mixture-model analysis to independently estimate the proportion of guesses and the precision of non-guess responses. We find that the improvements in performance conferred by retrospective attention are overwhelmingly determined by a reduction in the proportion of guesses, providing strong evidence that attracting attention to the target’s location after its disappearance increases the likelihood of perceiving it consciously.

## Introduction

What is the role of attention in conscious perception? This question is central in current discussions of the neural mechanisms of conscious perception [1–3]. Some authors propose that conscious perception is entirely determined during the build-up of representations within sensory areas, and that, although attention can modulate this process, it is not part of the core mechanisms of awareness [3, 4]. In contrast, other authors propose that conscious perception arises when and only when sensory representations are broadcast, shared and maintained within a wider network of cortical regions, including supra-modal areas [1, 5]. In this latter view, attention would act as a gatekeeper that mediates this broadcasting event. This second proposition leads to a strong prediction: if a sensory representation initially fails to become conscious, it should still be possible to promote this representation into awareness by orienting attention towards its residual sensory trace [6], even after the stimulus itself has disappeared.

Cueing attention after a visual display has classically been used in “iconic memory” experiments, where each display contains several high-contrast items, for example an array of letters [7, 8]. These experiments show that, although participants are limited in the number of letter identities they can report from a briefly presented array (no more than 3 or 4), cueing attention to one specific row within one second after the display can still improve how well these cued letters are recalled. A classical interpretation of this effect is that post-cued attention can bias which items are transferred to working memory. In such protocols it is difficult to assess whether conscious perception itself is affected by post-cueing. Specifically, when the number of items presented exceeds working memory capacity, one can argue that what is reported is less than what has been consciously perceived. In other words, in these type of experiments there may be a dissociation between the content of conscious perception and the content of conscious access (i.e. the representations that are present in working memory and can be reported).

In order to test our prediction that perception itself can be influenced by retrospective attention, we developed a protocol where we ask participants to report a single Gabor patch at threshold contrast. In this case, the stimulus does not exceed working memory capacities and report should faithfully reflect conscious perception. In a series of experiments we tested the influence of retrospective attention (or “retro-cueing” [9]) on the perception of this single Gabor patch [10]. We showed that attracting exogenous attention to the stimulus location after its disappearance improved objective orientation and detection sensitivity (*d*’) as well as subjective visibility [10], suggesting that retrospective attention can indeed improve conscious perception.

While this effect could be taken as evidence that retro-cueing elicits a discrete transition to conscious access, and thus from no conscious perception to conscious perception, another possibility is that performance improves because retro-cueing affects the fidelity with which an already conscious content is maintained. Studies on working memory suggest that retrospective attention can prevent rapid forgetting of fine-grained information in displays with multiple high-contrast items [11, 12], so the same process could be at work in this retro-perception phenomenon. A two-alternative forced-choice (2AFC) task does not allow these options to be disentangled; changes in the number of seen trials or changes in the quality of a conscious representation produce similar changes in performance for forced choice.

**Fig 1:**
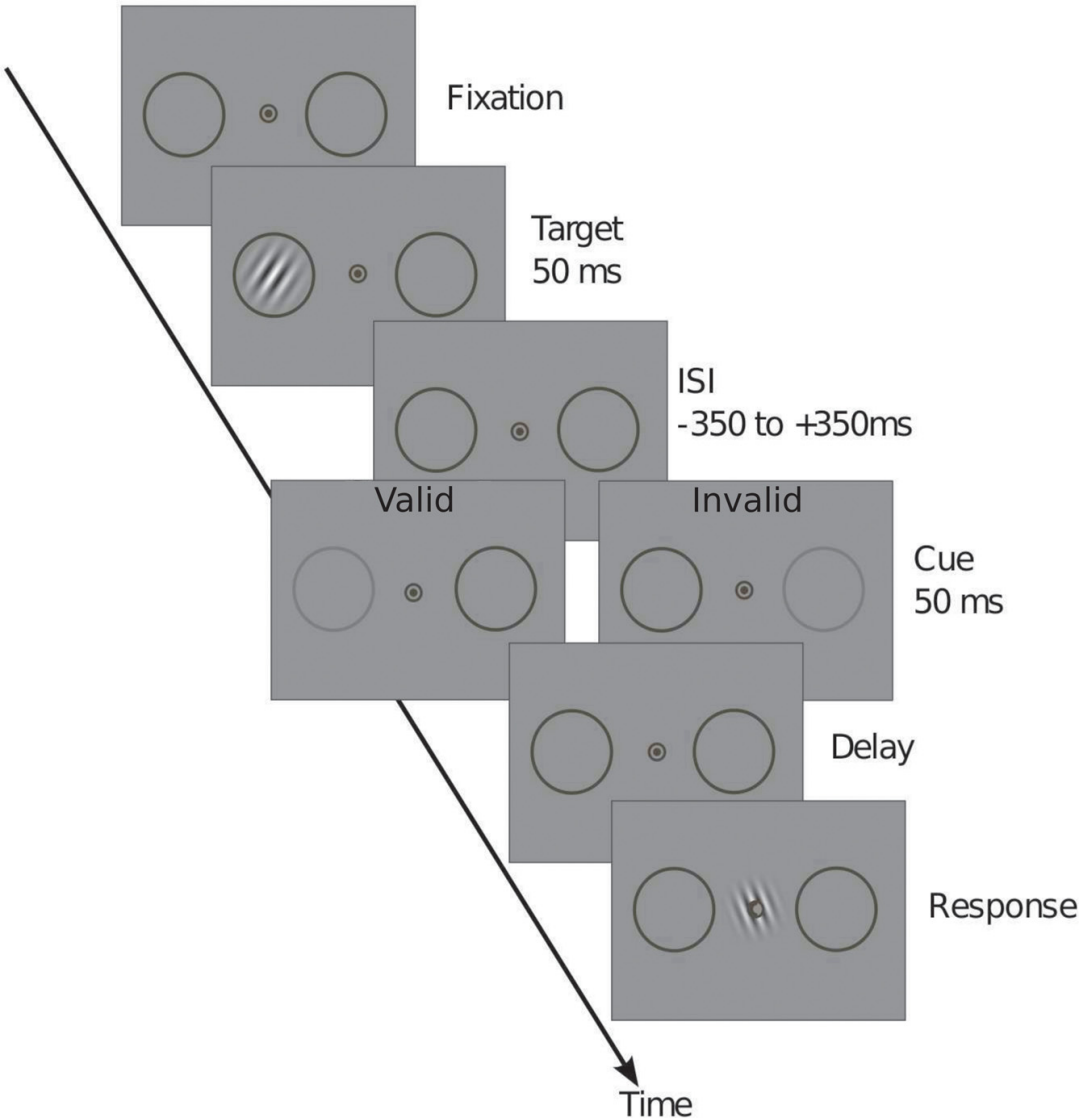
Experimental design. A target appeared in either one of the circular placeholders and was preceded or followed by a pre or retro cue in the form of a brief dimming of one of the placeholders. Subjects reported the orientation of the target using the central Gabor patch. On the response screen, a report cue (thickening of one side of the fixation circle) indicated where the target had been presented so that there was no location uncertainty at the time of the response. Note: stimuli are not to scale on this representation.

**Fig 2:**
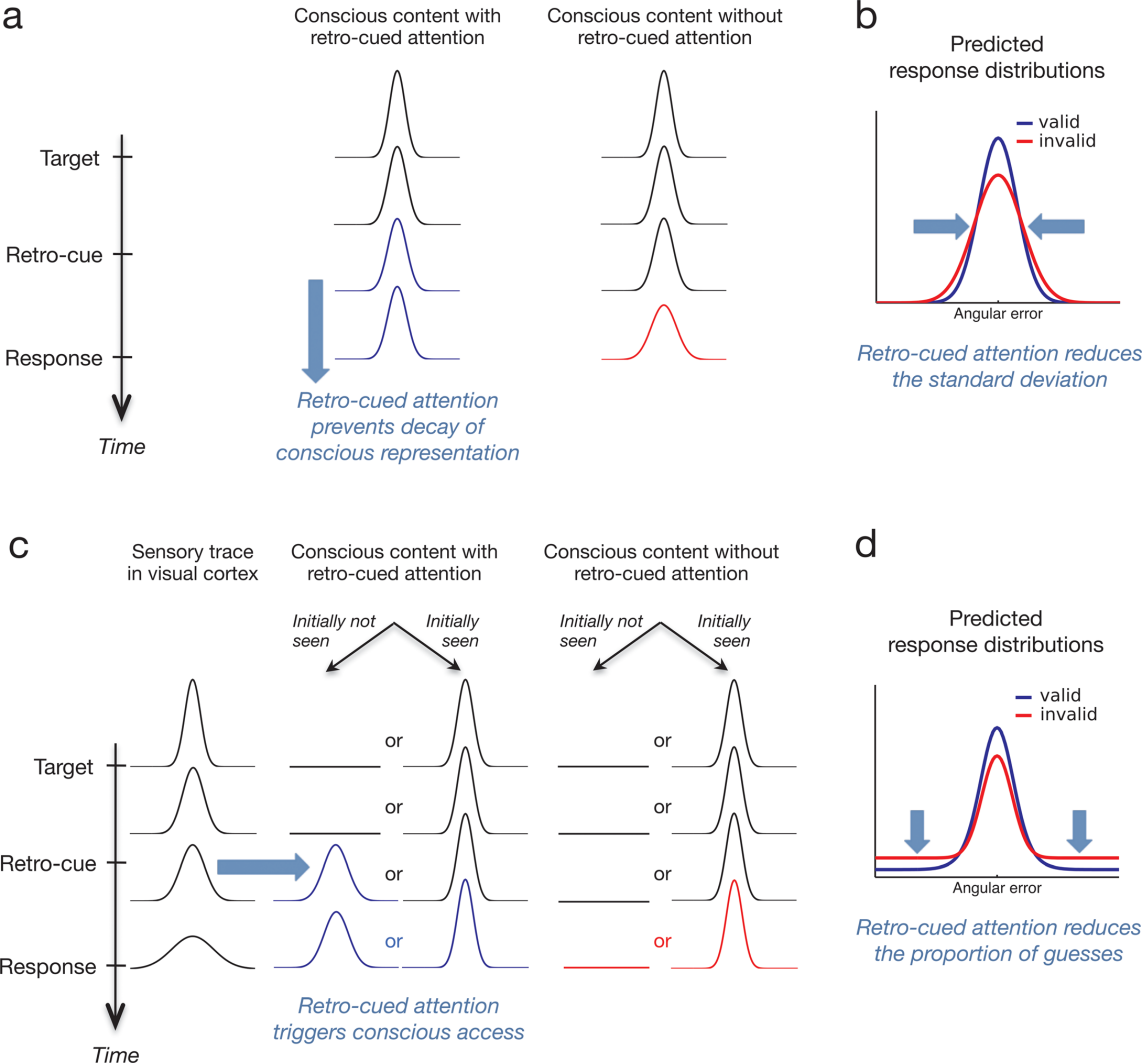
Hypotheses and predictions. According to a first hypothesis (a) retro-cued attention prevents the decay of an existing conscious percept. In this proposition, even when the target is conscious, in the absence of retro-cued attention the precision of this conscious representation decays slightly with time (right column). When retro-cued attention is focused on the target’s location (left column), it would prevent this slight decay of the conscious representation. This hypothesis thus predicts that the precision of the response on target’s orientation should be increased for valid retro-cues (blue curve) compared to no cues or invalid retro-cues (red curve) (b). The alternative hypothesis (c) is that retro-cued attention triggers conscious perception on trials where the target would otherwise have been missed. In this proposition, the target is not always consciously accessed, and thus not always consciously seen following its presentation (right column), but it always leaves a sensory trace in the visual cortex (left column). On trials were the target initially failed to reach conscious access, retro-cued attention at the target’s location could still promote the remaining sensory trace in visual cortex at this location to be consciously accessed (middle column). This hypothesis predicts that valid retro-cues (blue curve) should decrease the number of guesses compared to no cues or invalid retro-cues (red curve) (d). It also predicts a decrease in the precision of the accessed information: indeed valid retro-cues trigger conscious access to a degraded sensory trace on trials that otherwise would have counted as guess. Thus, less precise representations get included in the standard deviation estimate.

The present study directly tests these two options through the use of a finer grained measure of perceptual content: a continuous, stimulus-matching task or “reproduction task” (Fig 1). Instead of choosing between two options (correct orientation versus orthogonal orientation), subjects were instructed to continuously adjust the orientation of a probe in order to match the orientation of the preceding target. Previous literature shows that the response distributions in such reproduction tasks can often be accurately described by a mixture between a Gaussian distribution around the target’s true orientation with a certain standard deviation, and a uniform distribution, due to trials where subjects guessed, i.e. responded in the absence of information about the target’s actual orientation [13, 14]. A mixture model analysis of these distributions allows for separate estimates of the proportion of “guess” trials in which the target was not consciously accessed, and the precision of the consciously accessed representations. The two accounts of retro-perception make opposite predictions regarding these measures (Fig 2). In the first account (Fig 2A and 2B), retro-cued attention may prevent the typical loss of precision of the target with time for targets that are already in awareness. Consequently valid retro-cues (same side as the target) should increase the precision of reported target orientation relative to invalid cues, without affecting the percentage of guesses (Fig 2A and 2B). Alternatively, in the second account (Fig 2C and 2D) retro-cued attention may act on targets that have not reached awareness and bring their initially unconscious sensory trace into awareness. In this case, the frequency of guesses should decrease with valid retro-cues compared to invalid ones while the precision of responses should be unaffected (Fig 2C and 2D).

## Materials and Methods

### Participants

The number of participants was fixed to twenty prior to the experiment, based on our previous observation and replications of the retro-perception effect [10]. Twenty participants between the ages of 18 and 32 took part in the study, each exhibiting normal or corrected-to-normal vision. Of these, three were excluded because they failed to converge on a stable contrast threshold during the initial staircase, or because they failed to perform above chance in all conditions. The 17 remaining subjects (9 women, 8 men) had an average age of 23.7 years ±2.1. All participants gave informed consent in writing prior to participation, and the Université Paris Descartes Review Board, CERES, approved the protocols for the study in accordance with French regulations and the Declaration of Helsinki. Participants received a compensation of 10€ per hour for their time.

### Apparatus and Stimuli

Stimuli were generated and responses recorded using the Psychophysics Toolbox for Matlab [15]. Stimuli were presented on a CRT monitor (Sony Trinitron GDM-F520). Refresh rate was 60 Hz and screen resolution was 1280 by 1024 pixels. Participants were seated 60 cm away from the monitor, in a dimly lit room. Eye fixation was monitored and recorded using an Eyelink 1000 (SR Research Ltd., Osgoode, Ontario, Canada). We verified that subjects maintained fixation during the majority of trials. This was determined by counting the number of trials during which the mean fixation exceeded 1 degree of eccentricity from the central fixation point (the border of the placeholders were at 3 degree on each side). On average, subjects exceeded this threshold on 0.3% of the trials.

Stimuli (Fig 1) were presented on a gray background (12 cd/m^2^), and participants were told to fixate a small black circle at the center of the screen (.6° in diameter). Two larger black circles (2.4° in diameter) were always present bilaterally, with their centers positioned 4° to the left and right of central fixation, and served as placeholders for the two possible target positions. They also provided a means for attentional cueing, as introducing a brief decrease in one of the placeholders' contrast produced an attention-grabbing flash.

Targets were Gabor patches subtending 2° in diameter (2 cycles per degree with a randomized phase; Gaussian envelop with 1° full width at half maximum) and were presented in one of twelve orientations spanning 7.5°to 172.5° in increments of 15°. The contrast of the target was determined for each individual using a staircase procedure that converged on a hit-rate of 80% (proportion of trials with an absolute angular error smaller than 45°).

Each trial began with the onset of a dot at the center of the fixation circle. Following a random delay between 500 ms and 900 ms, a target was presented for 50 ms within either of the placeholders. A brief reduction in the contrast of one of the placeholders, turning from black to dark gray (6 cd/m^2^) for 50 ms, drew attention to the side of the target (valid cue) or the opposite side (invalid cue). This attentional cueing could take place before (SOA -100 ms) or after the target (SOA 100 ms or 400 ms). Each experimental block of 156 trials contained 12 trials where no cue was presented. A response screen appeared following another 500 to 900 ms delay, comprising a response cue in the form of a thickening on one side of the central fixation circle that indicated where the target had appeared, so that there was no uncertainty about the target’s location at the time of the response. The response screen also included a response Gabor patch presented at fixation and subtending 2 degrees of visual angle that subjects used to reproduce the target’s remembered orientation. Its parameters were the same as the target except that its contrast was 100%, its sinusoidal phase was fixed at .5 radians, and its initial orientation was random.

The participant's task was to reproduce the remembered orientation of the target by freely and continuously varying the orientation of the response patch using the mouse. A small black dot above the response patch indicated the mouse position on the screen. Subjects were not limited in their response times. They indicated their final choice with a left click. Subjects were instructed to always provide a response, and guess in the event that they had not seen the target. Feedback was provided at the end of each block in the form of percentage of hits (a response deviating more than 45° relative to the target orientation was considered as a “miss”).

### Procedure

The experiment consisted of two or three staircase blocks of 80 trials and 8 experimental blocks of 156 trials each. The “staircase” blocks consisted of a psychometric staircase function (weighted up down procedure [16]) that converged on a hit-rate of 80% (proportion of trials with an absolute angular error smaller than 45°) [17]. Staircase blocks were identical to their experimental counterparts with two exceptions: (1) no cues were presented and (2) target contrast initially began at 100% and was decremented/incremented as a function of the correctness of the previous response (an absolute angular error smaller than 45° was considered as a correct response). In exceptional cases, a third staircase was performed to help stabilize performance (2 subjects).

In the standard experimental block, all targets were presented at the contrast for which the staircase function predicted 80% hit-rate in the absence of cueing (contrast was on average 3.38% ± .72%). At the end of each block, the participants received feedback in the form of their overall hit rate (percent of trials with angular error inferior to 45°). For four participants, the target’s contrast had to be readjusted between experimental blocks (once for two subjects, twice for one subject and three times for one subject) because the overall hit rate had become too high (> 90%) or too low (< 70%). Responses were collected over eight blocks of 156 trials resulting in a total tally of 192 trials for each Validity x SOA condition and 96 “no-cue” trials per subject.

### Analysis

#### Overview of analysis steps

The main question that we wish to answer is whether retro-cueing affects (i) the probability that subjects have conscious access to information about a past stimulus, (ii) the precision of this information, or (iii) both. To answer this question, we fit a range of plausible models to our data and examine the parameter estimates of the model that best accounts for the data. Before we fit the models, we remove bias from our data caused by the oblique effect [18]. All steps are described in detail below.

#### Bias correction

For each trial, the orientation of the subject's response was subtracted from the target's true orientation, yielding the angular error. Biases in angular error varied across target orientations due to oblique effects [18]. After verifying that the magnitude of this oblique effect was unaffected by our experimental conditions (see S1 Fig), we normalized our data in the following way. This bias estimation and correction was performed independently for each participant. For each participant, we took the median angular error as a function of the 12 possible target angles across all experimental conditions as a first estimate of the biases profile. Since oblique effect biases were symmetrical around the vertical and horizontal meridians for each subject, we further averaged the absolute bias across symmetrical angles and replaced the initial estimates with this average, correctly signed. This bias estimate for each target angle was subtracted from the corresponding error distribution, thereby yielding error distributions centered around zero for all target angles.

#### Basic mixture model

**Fig 3:**
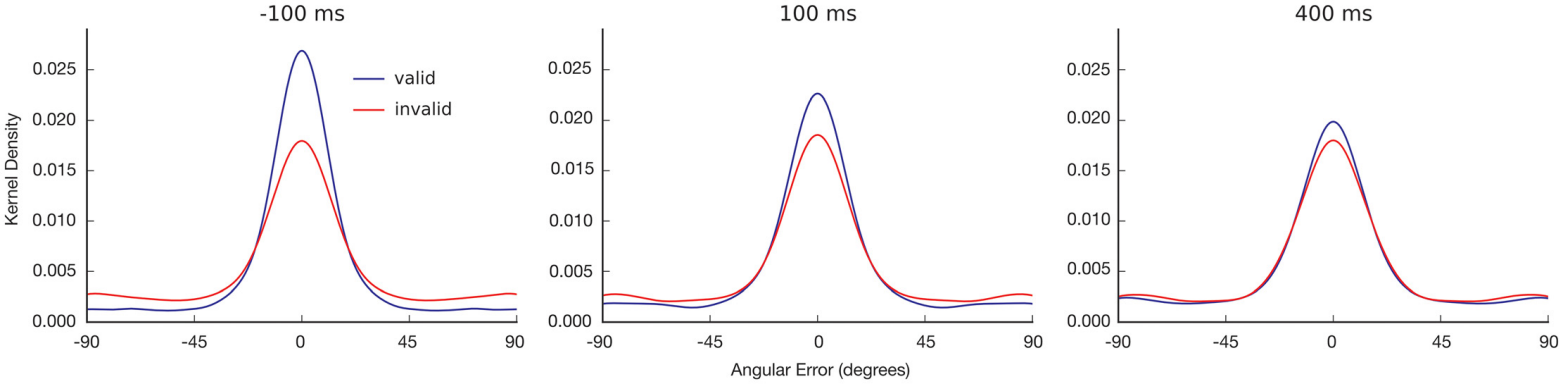
Observed response distributions. Distributions of the angular response errors around the target’s true orientation (kernel density estimation) for valid and invalid cues at the three different SOAs: pre-cues (-100 ms, left panel) or retro-cues (100 ms and 400 ms, middle and right panels). A reduction in the uniform component (*P*_guess_) is apparent across SOAs as a difference between the valid and invalid distributions in the extrema of the curve.

We hypothesize that the distribution of a subject’s orientation judgment errors (Fig 3) reflects two kinds of trials: trials in which orientation information was consciously available and trials in which such information was not available [14]. In the first type of trial, errors are expected to follow a Von Mises distribution (the circular equivalent of a normal distribution) that is centered on the target’s true orientation. The width of this distribution reflects the average precision with which the orientation was remembered: a narrower distribution means that the orientation was on average remembered with higher precision. In the second type of trial, responses are expected to be pure guesses, thus producing a uniform error distribution. The predicted error distribution, i.e. the probability of producing an angular error *x*, is thus of the form:

$$p(x\;|\;P_{guess},\;\kappa )=\;P_{guess}\frac{1}{2\pi }+\left(1-\;P_{guess}\right)\frac{1}{2\pi I_{0}\left(\kappa \right)}e^{\kappa \;\text{cos}\left(x\right)}$$ (1)

where

- The first term specifies the (uniform) guessing component and the second term specifies the (Von Mises) non-guessing component;
- *x* is the angular error (in radians);
- *P*_guess_ is the proportion of guess trials (a free parameter);
- *κ* is the concentration parameter of the Von Mises distribution; this free parameter can be interpreted as the precision of the memory (higher *κ* produces a narrower error distribution);
- *I*_0_(·) is the modified Bessel function of the first kind of order 0 (the formula is provided as supplementary information)

#### Factorial design of 4 mixture models for model selection

The basic mixture model specified above assumes that memory precision is a fixed quantity throughout the experiment. However, several studies have found that, in classical working memory experiments where each display contains several high-contrast items, working memory errors are often better accounted for by models in which working memory precision varies across items and trials [19–21]. Moreover, such variable-precision models do not necessarily need a guess rate to successfully explain memory errors. To examine whether our data are best accounted for by an equal-precision (EP) or variable-precision (VP) model and whether or not a guessing component is required, we implemented a factorial model design with 2 factors (“variability in precision” and “guessing”) with 2 levels each (“absent” and “present”). This 2x2 design thus gives rise to the following 4 models:

1. Equal precision without guessing
2. Equal precision with guessing (i.e. the basic mixture model described above)
3. Variable precision without guessing
4. Variable precision with guessing

If we find that the data are best accounted for by a model without a guessing component, we should conclude that retro-cueing can only affect precision (or variability in precision) and not the probability with which a subject has conscious access to information about a past stimulus. If, on the other hand, we find that the data are best accounted for by a model with a guessing component, then we can analyze the parameter estimates to examine the effect of retro-cues on recall precision and the guess rate.

Following previous work [19, 20], we model variability in precision across trials by using a gamma distribution. Defining precision as the concentration parameter of the Von Mises distribution, *κ*, the predicted distribution of orientation errors in the VP-with-guessing model is thus specified as

$$p\left(x\;|\;P_{guess},\;\kappa \right)=\;P_{guess}\;\frac{1}{2\pi }+\left(1\;-\;P_{guess}\right)\int \frac{1}{2\pi {\text{I}}_{0}\left(\kappa \right)}e^{\kappa \;\text{cos}\left(x\right)}\;\text{Gamma}(\kappa \;|\;\bar{\kappa },\;\tau )d\kappa$$ (2)

where $\text{Gamma}(\kappa \;|\bar{\kappa },\;\tau )$ is the Gamma distribution with a mean $\bar{\kappa }$ and shape parameter *κ*. The predicted error distributions of the EP and VP models without guessing are identical to the models specified in Eqs (1) and (2), respectively, but with *P*_guess_ fixed to 0.

#### Model fitting

We divided the data of each subject into 7 subsets (2 cueing conditions, valid or invalid, times 3 SOAs plus a no-cue condition). We fit all models separately to each of the 7 subsets. Fitting was done using Matlab’s fminsearch function to find the maximum likelihood parameter values.

#### Statistics

When reporting the ANOVAs “F”, we report corrected degrees of freedom using Greenhouse-Geisser.

## Results

### Model-free analyses

**Fig 4:**
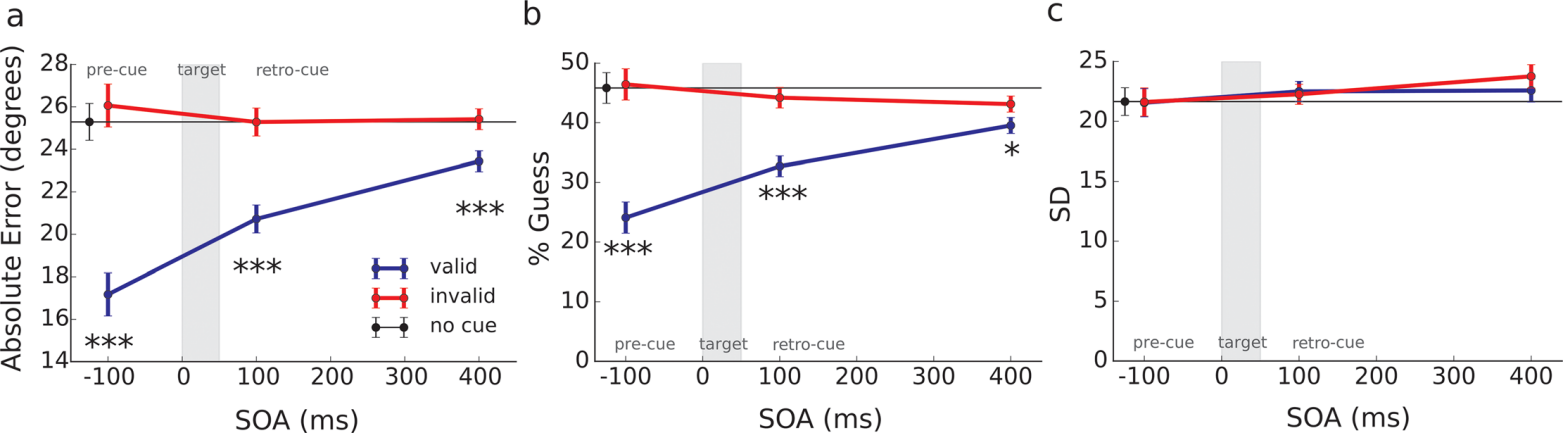
Angular error and parameter estimates. Effect of cue validity and SOA on mean absolute response error (a), on percentage of guesses (parameter *P*_guess_ of the model) (b), and on standard deviation (SD) (c). Error bars represent standard error of the mean effect size. For “no cue” trials, error bars represent the standard error of the mean.

Both pre and retro-cueing improved performance in reporting the target’s orientation, as reflected in the response distributions (Fig 3) and in the average absolute angular error around the target’s true orientation (Fig 4A). We analyzed how the average absolute angular error varied as a function of our experimental conditions (Fig 4A) using a repeated-measures analysis of variance on Validity x SOA (2x3). Participants were more accurate in reproducing the target’s orientation (decrease in absolute angular error) on trials where the cue attracted their attention to the side where the target appeared (valid cue) compared to trials where the cue was on the opposite side (invalid cue), *F*(1, 16) = 79.96, *p* < .001, *d* = 1.57. This was true for cues presented before the target, *t*(16) = -.79, *p* < .001, *d* = 2.28, as expected from the classical literature on attention [22–24], and also for cues presented after the target disappeared: SOA 100 ms, *t*(16) = -6.10, *p* < .001, *d* = 1.21, and SOA 400 ms, *t*(16) = -3.60, *p* < .005, *d* = .63. The effect of SOA was significant, *F*(2, 32) = 18.36, *p* < .001, as was the interaction between validity and SOA, *F*(2, 32) = 37.56, *p* < .001. These results replicated the effects observed in previous retro-perception experiments with this new measure of angular error [10].

We found no significant difference in average error on trials that were invalidly cued versus those where the cue was absent. This comparison with the “no-cue” baseline condition revealed that the above effect of validity on task accuracy was due to a benefit of valid cueing rather than a cost of invalid cueing.

### Model selection

To obtain insight into the statistical nature of the error distributions, we fit 4 different mixture models (see Methods) to the data of each subject. For each of these 68 model fits (17 subjects times 4 models), we computed the Bayesian Information Criterion (BIC), which is a measure of how well a model accounts for the data, taking into account the number of free parameters [25]. For 16 out of 17 subjects, the EP(equal precision)-with-guessing model was the preferred model, outperforming the runner-up model with an average BIC difference of 19.5±4.2 (mean±sem). For the remaining subject, the VP(variable precision)-without-guessing model was the preferred model. However, the BIC difference with the EP-with-guessing was only 0.6, which is negligible. Hence, a guessing component is important to account for the data, but variability in precision across trials is not required. This observation contrasts with recent studies of visual working memory using displays with several high-contrast items where estimation error data are better accounted for by variable-precision models [19, 20]. However, those studies all used set sizes larger than 1. If variability in memory precision is caused by an inability in dividing mnemonic resources exactly equally across multiple items, then it is not surprising that we do not find evidence for such variability in our study.

### Precision versus guessing for the selected model

Having identified the best model (the standard mixture model, i.e. equal precision with guessing), we investigated whether the improvement observed with pre and retro-cued attention was due to a decrease in either the proportion of guesses or the standard deviation (Fig 3). In the absence of cueing the proportion of guesses was around 45% (Fig 4B). Both pre and retro-cueing to the target’s side reduced the number of trials in which subjects guessed their responses, as evidenced by a reduction in *P*_guess_ for valid trials relative to invalid trials, *F*(1, 16) = 86.70, *p* < .001, *d* = 1.45. This was once again true for trials in which the cue preceded the target, *t*(16) = 8.49, *p* < .001, *d* = 2.03, and when the cue followed the target by 100 ms, *t*(16) = 6.64, *p* < .001, *d =* 1.23, or even by 400 ms, *t*(16) = 2.67, *p* = .017, *d* = .42. We found a significant main effect of SOA, *F*(1.76, 28.23) = 9.27, *p* = .001, and a significant interaction between validity and SOA, *F*(1.51, 24.09) = 27.94, *p* < .001, mirroring the pattern of results observed for angular error.

In contrast, cue validity did not significantly affect the precision of report for seen trials, as reflected by the standard deviation (SD) parameter (Fig 4C), *F*(1.00, 16.00) = .44, *p* = .250. There was a modest increase of SD with SOA, *F*(1.85, 29.49) = 3.90, *p* = .034. No interaction was found between validity and SOA, *F*(1.71, 27.28) = .489, *p* = .589.

## Discussion

Our aim here was to test the prediction that retrospective attention can trigger conscious perception. We asked participants to report a single target Gabor patch, shortly after it has been presented (less than a second), with no uncertainty on where it has been presented (thanks to a response cue). In this setting, if participants fail to report this target, it is reasonable to assume that they also failed to perceive it consciously. Conversely, improved performance should reflect improvement in conscious perception.

The present results confirm our previous observation of a retroactive effect of attention on conscious perception, a phenomenon we call “retro-perception” [10]: although the target was a single Gabor patch at threshold, attracting exogenous attention on its location 100 ms or 400 ms after its presentation substantially improved participants’ ability to reproduce its orientation (Fig 4A). In our previous studies, results on subjective visibility suggested that retro-attention triggered discrete transitions in conscious access, and thus in conscious perception (Fig 4 in [10]). In the present study we formally tested this proposition using a continuous orientation matching task and a mixture model analysis.

Although mixture model analyses have mainly been used in working memory experiments with several high contrast items [26, 27], Asplund and colleagues recently used this method to confirm that the attentional blink, which is known to impair perception, is not due to a degradation of the sensory representation of the “blinked” stimulus but to a discrete blocking of conscious access to that information (increase in the number of guesses) [13]. This was an elegant way to corroborate, using an objective measure, observations that were initially made using a subjective visibility measure [28, 29]. Here we adopted the same strategy to probe the effect of retrospective attention on precision and guessing. A comparison of four plausible models confirmed that the standard mixture model (equal precision across trials plus guessing) was the one that accounted best for the response distributions obtained in the present study. The parameters estimated from this model showed a very clear-cut pattern whereby the benefits of pre or retro-cued attention were accounted for by a reduction in the number of “guesses” (Fig 4). By contrast, the precision of representation was not affected by whether the cue was valid or invalid (Fig 4C).

These results rule out the hypothesis that the retro-perception effect stems from a memory rather than a perceptual effect; if retro-perception prevented a rapid decay of seen representations in memory, we should have observed an improvement of precision in valid retro-cue trials compared to invalid or no-cue trials (see hypotheses and predictions in Fig 2). We find no evidence for such improvement. So, in contrast with the widely-held assumption that events occurring after the disappearance of a stimulus can only affect post-perceptual processes such as decision or working memory [30–33], the present results show that retro-cued attention can also directly affect whether the stimulus is seen or not.

Our results provide strong support for models of consciousness according to which conscious perception arises when and only when representations held in sensory cortex are broadcasted and maintained within a “global workspace” that includes higher-level cortical areas [1, 34, 35]. In such models conscious access and conscious perception are tightly linked and these models suggest that attentional selection acts as a gatekeeper for such broadcasting mechanisms. The present study validates a very strong and counter-intuitive prediction of these models: even when a stimulus initially fails to be perceived consciously, inducing a reactivation of the associated sensory trace by attention can promote it to awareness. In other words, attention can cause conscious perception after the stimulus has disappeared. The present observations also support the notion of “preconscious representations” that we developed earlier [1]: in the present experiment, when a target initially fails to become conscious, it is preconscious in the sense that its conscious fate is still uncertain, since retro-cueing can still promote it to conscious access and conscious perception.

Here we chose an experimental setting in which, by construction, behavioral report should faithfully reflect conscious perception. In iconic memory experiments, by contrast, the link between report, conscious access and conscious perception is less straightforward and still very much debated [36, 37]. In these experiments the number of items, and more generally the complexity of the display, exceeds working memory capacity, thus opening the possibility of a dissociation between what is perceived and what can be reported. The beneficial effect of post-cueing in iconic memory experiments has been taken as an indication of such a dissociation: since what is reported can be flexibly influenced by a post-cue, this might indicate that the initial percept is richer than the subset that is extracted for report. This interpretation of the iconic memory phenomenon has become a core argument in favor of the existence a form of conscious perception, called phenomenal consciousness, which may greatly exceed the scope of details available to conscious access [36, 38]. However, this interpretation relies on the assumption that what comes after the stimulus cannot induce conscious perception of elements that were not initially perceived consciously. The current results show that this assumption is not supported: here a discrete transition to conscious perception was induced by orienting attention after the stimulus had disappeared. As such, one cannot exclude the possibility that retro-perception mechanisms are also at play in iconic memory experiments, and hence account or partly account for the beneficial effect of post-cueing, as suggested by alternative interpretations of the iconic memory phenomenon [39].

In summary, the present results show that conscious access displays a discrete component, and that attentional cueing can gate this discrete transition to conscious access and conscious perception, even after the stimulus is gone. This provides strong evidence for the hypothesis that attention plays a crucial role in conscious perception.

## Supporting Information

S1 Fig Bias profile and oblique effect. The graph represents the median of signed angular error relative to the target’s true orientation for each target orientation and each experimental condition averaged accross participants. While the absolute angular error gives us an estimate of the dispersion of the errors, the median of signed errors indicates the center of the error distribution. When this center is 0, it means that there is no bias in the perception of the target’s orientation. Here we see the classical “oblique effect” bias as a deviation from 0 for target orientations close to the horizontal or vertical. This oblique effect profile did not vary significantly across experimental conditions.
(TIF)

S1 Text Formulae. (PDF)
