# Supplementary figures and images for: Retrospective Attention Gates Discrete Conscious Access to Past Sensory Stimuli
Source: PLoS One. 2016 Feb 10;11(2):e0148504. doi: 10.1371/journal.pone.0148504 (PMC4749386; doi:10.1371/journal.pone.0148504)

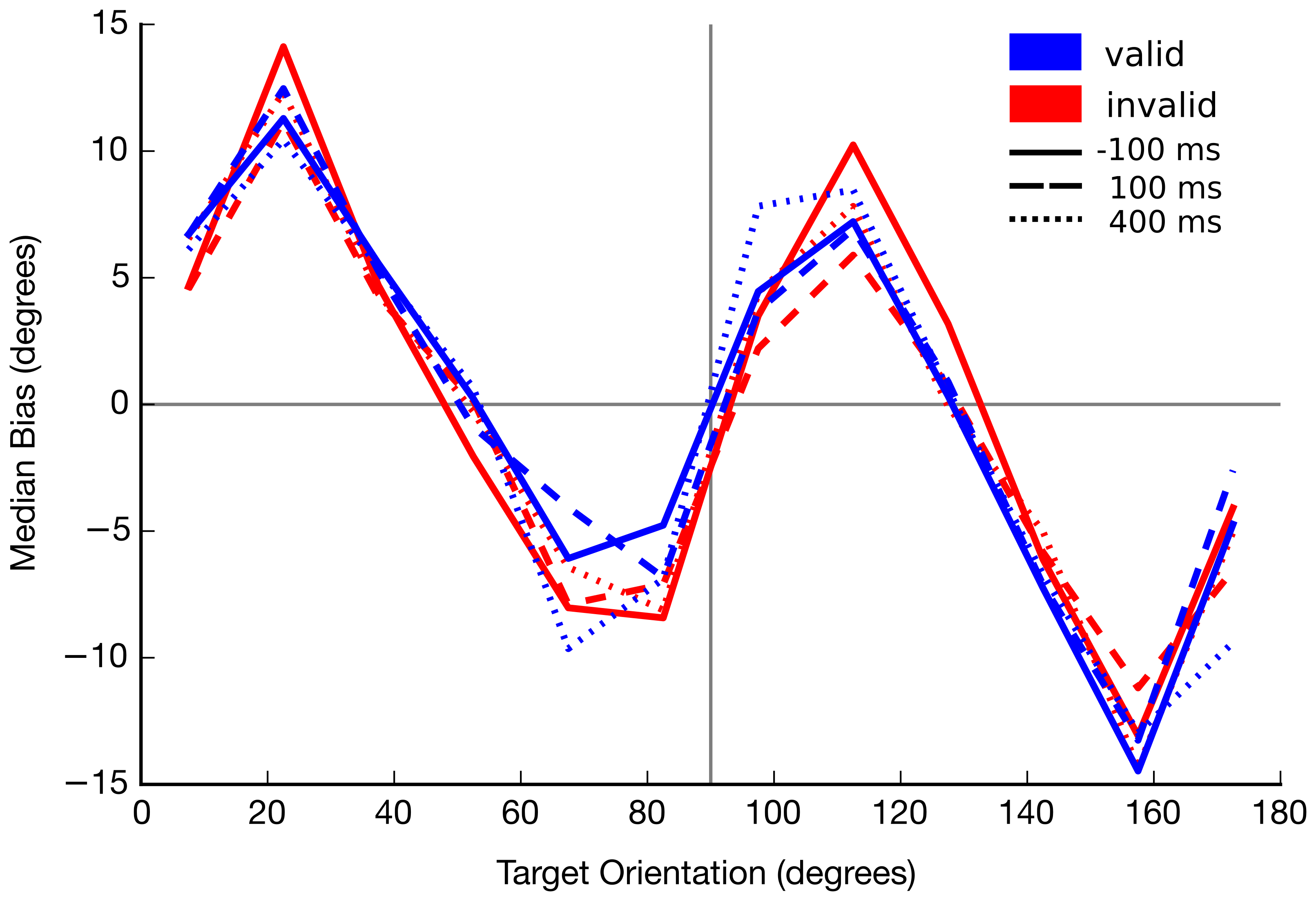

Supplement: S1 Fig — The graph represents the median of signed angular error relative to the target’s true orientation for each target orientation and each experimental condition averaged accross participants. While the absolute angular error gives us an estimate of the dispersion of the errors, the median of signed errors indicates the center of the error distribution. When this center is 0, it means that there is no bias in the perception of the target’s orientation. Here we see the classical “oblique effect” bias as a deviation from 0 for target orientations close to the horizontal or vertical. This oblique effect profile did not vary significantly across experimental conditions. (TIF) [file pone.0148504.s001.tif]
