# Supplementary material for: Retrospective Attention Gates Discrete Conscious Access to Past Sensory Stimuli
Source: PLoS One. 2016 Feb 10;11(2):e0148504. doi: 10.1371/journal.pone.0148504 (PMC4749386; doi:10.1371/journal.pone.0148504)
Supplement: S1 Text — (PDF) [file pone.0148504.s002.pdf]

Supplementary Information

Formulas

1. Bessel equation of order v:

$$I_v(z) = \left(\frac{z}{2}\right)^v \sum_{(k=0)}^{\infty} \frac{\left(\frac{z^2}{4}\right)^k}{k!\,\Gamma(v+k+1)}$$

where Γ is the gamma function.

2. The conversion of the precision parameter κ to standard deviation (SD) follow the equation:

$$SD = \sqrt{-2 \cdot \ln \frac{I_1(\kappa)}{I_0(\kappa)}}$$
